# Extensive intrathoracic and intraperitoneal splenosis mimicking mesothelioma: a case report

**DOI:** 10.1186/s13256-022-03288-9

**Published:** 2022-02-19

**Authors:** Bandik Föh, Malte Maria Sieren, Marcus Both, Marcus Seeger, Rainer Günther

**Affiliations:** 1 Medical Department I, University Hospital of Schleswig-Holstein, Building A, Ratzeburger Allee 160, 23562 Lübeck, Germany; 2 Institute of Nutritional Medicine, University of Lübeck, Ratzeburger Allee 160, 23562 Lübeck, Germany; 3 Department of Radiology and Nuclear Medicine, University Hospital of Schleswig-Holstein, Ratzeburger Allee 160, 23562 Lübeck, Germany; 4 Department of Radiology and Neuroradiology, University Hospital of Schleswig-Holstein, Arnold-Heller-Straße 3, 24105 Kiel, Germany; 5 Hepatology Division, Department of Internal Medicine I, University Hospital of Schleswig-Holstein, Arnold-Heller-Straße 3, Bd. C, 24105 Kiel, Germany

**Keywords:** Benign tumors, Case report, Contrast-enhanced ultrasound, Mesothelioma, Splenosis

## Abstract

**Background:**

Splenosis is the heterotopic autotransplantation of splenic tissue after severe splenic trauma and/or splenectomy. The epidemiology is elusive, but splenosis is frequently misdiagnosed as malignant tumors of gastrointestinal, gynecological, or hematological origin before the correct diagnosis is ultimately found. We herein report a rare case of combined, extensive intraabdominal and intrathoracic splenosis initially presenting as pleural mesothelioma.

**Case presentation:**

A 63-year-old Caucasian male presented with dyspnea and recurring thoracic pain. Initial X-ray and computed tomography scans showed disseminated intrathoracic and intraabdominal lesions. Consequently, thoracoabdominal mesothelioma or a polytopically metastasized cancer of unknown origin was suspected. A thorough examination of the patient’s medical history and contrast-enhanced ultrasound by a skilled examiner revealed the diagnosis of extensive abdominal and thoracic splenosis as a consequence of an abdominal gunshot wound with a ruptured diaphragm several decades earlier. Timely diagnosis by noninvasive measures prevented the patient from potential complications of harmful diagnostic procedures, including nuclear imaging and biopsies. The patient is currently treated for hepatitis C and chronic obstructive lung disease, whereas no specific treatment for splenosis is required.

**Conclusions:**

We present a case of rare intrathoracic and intraperitoneal splenosis mimicking mesothelioma. Contrast-enhanced ultrasound and thorough patient history were used for diagnosis and prevented this patient from having to undergo potentially harmful diagnostics. Splenosis can occur after splenic trauma and, consequently, needs to be considered as a rare differential diagnosis to malignant tumors of various origins when a matching patient history is obtained.

## Background

Splenosis is the acquired heterotopic autotransplantation of splenic tissue [1]. It should not be confused with accessory spleens, which is a common, benign, congenital condition that occurs in 10–44% of autopsy cases without a specific medical history [1]. Splenosis, on the other hand, is a comparably rare condition that typically occurs after severe splenic trauma and/or splenectomy. Although its overall epidemiology remains largely unclear, it is reported in 26–76% of patients with a corresponding history of splenic trauma [2, 3]. This divergence in case numbers is attributed to the rarely occurring symptoms that accompany the condition. Therefore, splenosis is mostly found incidentally after medical imaging reveals intraabdominal masses. Because splenosis typically presents with multiple, ectopic, intraabdominal foci, it is frequently misdiagnosed as metastatic cancer of gastrointestinal, hepatic, pancreatic, renal, or gynecological origin, leading to additional and potential harmful diagnostics [4]. In rare cases, splenosis might occur intrathoracically and has thus been misinterpreted as thoracic lymphoma, thymoma, or mesothelioma [4]. To raise awareness for the possibility of disseminated splenosis mimicking cancer of various origins, and to prevent misdiagnosis, we herein report a rare case of combined intraabdominal and intrathoracic splenosis that was diagnosed without invasive diagnostic measures.

## Case presentation

A 63-year-old Caucasian male presented to our hospital with mild dyspnea that had been present for years, and a recurring pain exacerbation in his left thoracic wall. His previous history included intravenous drug abuse, continued smoking for more than 30 years, and untreated hepatitis C infection. When similar symptoms had occurred a few months previously, X-ray scans performed at another hospital showed suspicious masses at the left costal pleura. Therefore, pleural mesothelioma was considered. However, the patient was discharged and did not follow-up on this diagnosis before presenting to us.

**Fig. 1:**
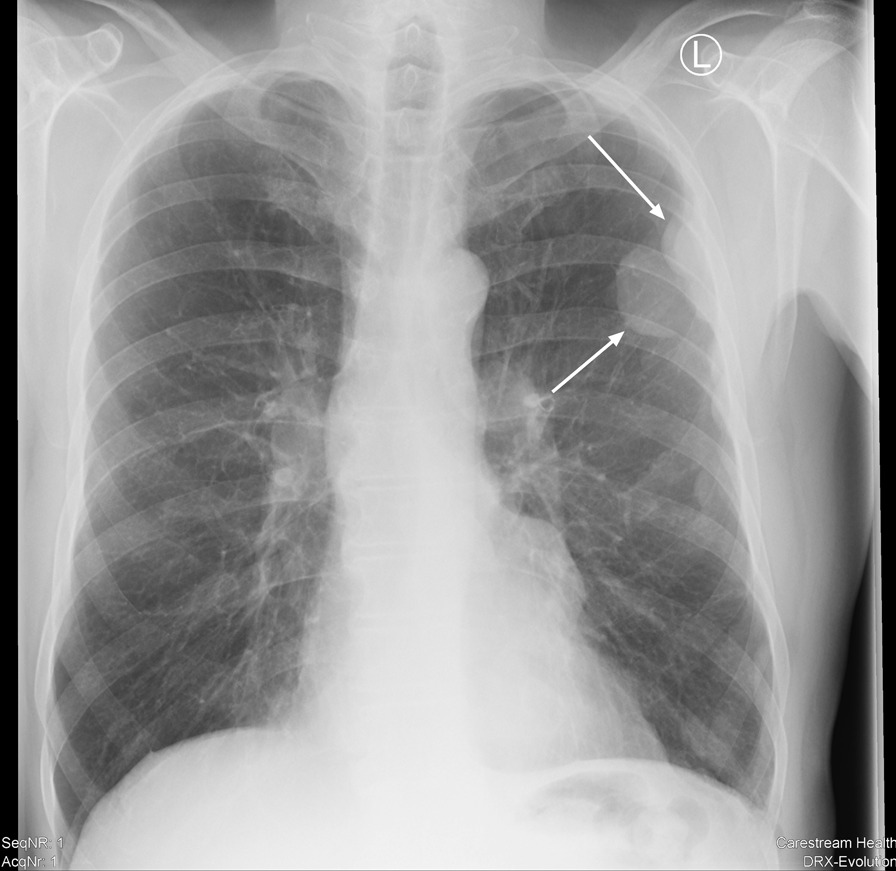
Chest X-ray at admission. White arrows indicate pleural masses, initially interpreted as possible mesothelioma

**Fig. 2:**
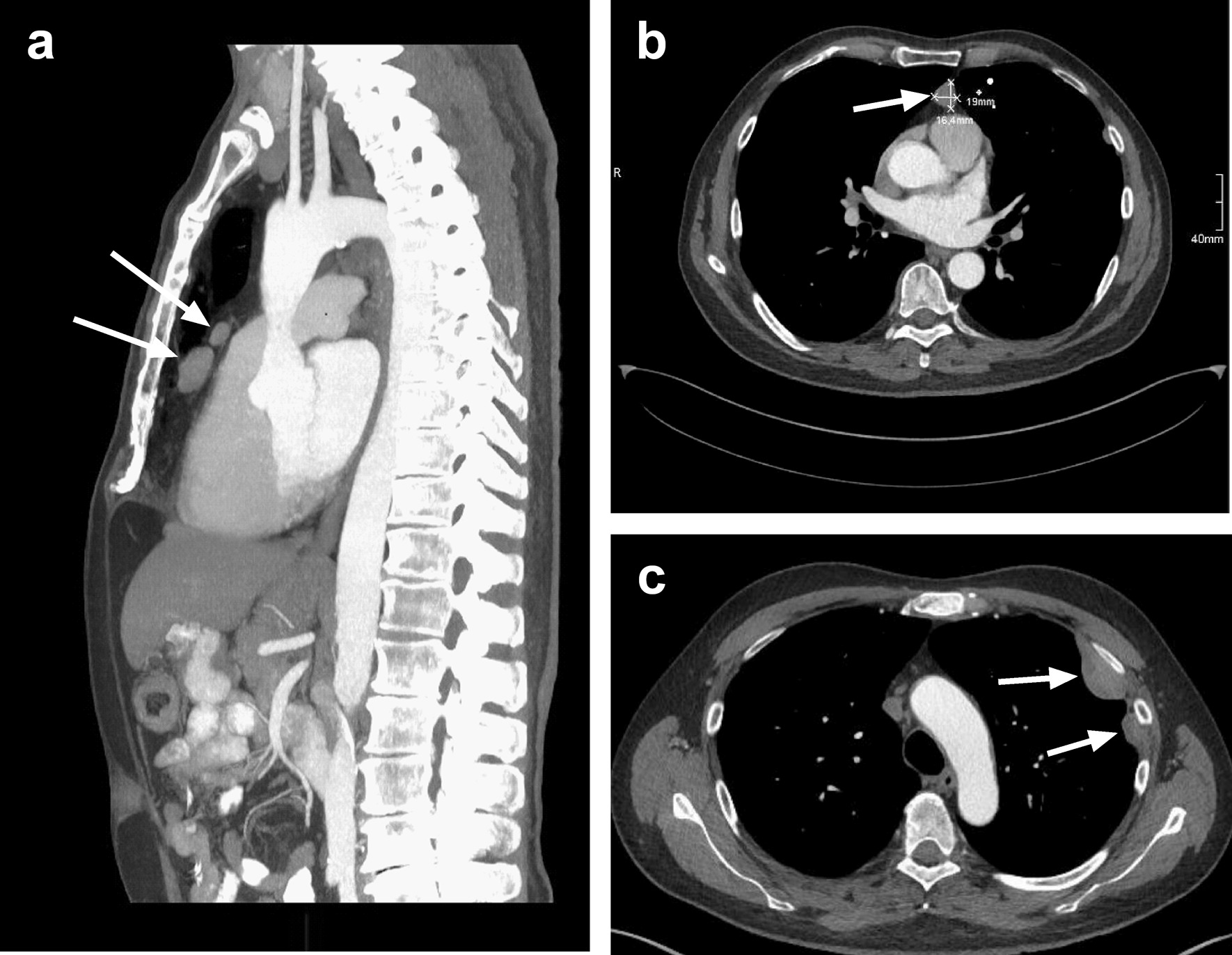
Contrast-enhanced thoracic CT scan (arterial phase). Sagittal (**a**) and axial planes (**b**, **c**) of the thoracic cavity. White arrows indicate masses in the anterior mediastinum (**a**, **b**) and at the left costal pleura (**c**)

After exclusion of myocardial ischemia, our X-ray scans showed the previously described lesions on the left costal pleura (Fig. 1). A subsequent contrast-enhanced computed tomography (CT) scan revealed two additional masses in the anterior mediastinum adjacent to the right atrium, measuring 2 and 4 cm in diameter (Fig. 2a, b) and recapitulated two lesions on the left costal wall measuring up to 5 cm in diameter (Fig. 2c). At this point, pleural mesothelioma, which had already been suspected elsewhere, was a plausible diagnosis.

**Fig. 3:**
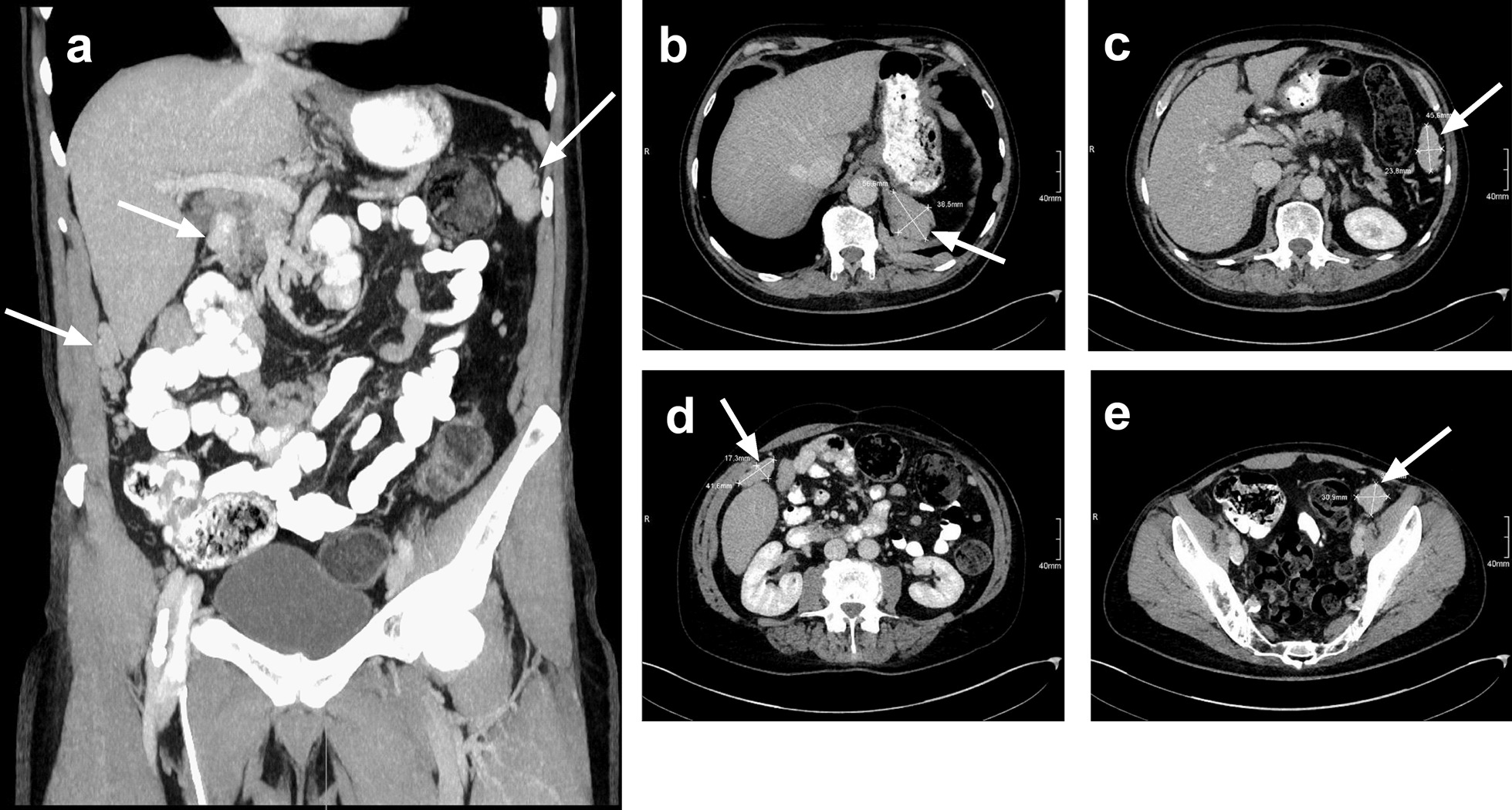
Contrast-enhanced abdominal CT scan (portal phase). Coronal (**a**) and axial planes (**b**–**e**) of the abdominal and pelvic cavities. White arrows indicate masses under the left diaphragm (**b**, **c**), between the abdominal wall and liver (**d**), and in the pelvic cavity (**e**)

However, multiple similar lesions were detected intraabdominally, whereas the spleen was missing (Fig. 3a). Specifically, two foci were found between the left diaphragm and the stomach (5.6 × 3.8 cm and 4.6 × 2.6 cm, Fig. 3b, c), between the liver and abdominal wall (4.1 × 1.7 cm, Fig. 3d), and in the small pelvic cavity next to the M. iliopsoas sinister (3 cm in diameter, Fig. 3e), among others. Curiously, the patient had to his knowledge never worked in an asbestos-polluted environment and the CT scans did not show any pleural effusions, which would typically be expected for mesothelioma [5]. Furthermore, the presence of abdominal masses is a possible, but not a common condition in mesothelioma [6]. Therefore, we considered a diagnostic biopsy to determine whether the lesions were metastases of a cancer of unknown origin.

However, we decided to first conduct an extensive interview with the patient that revealed an important, previously unknown part of his medical history: When asked for previous injuries, the patient remembered an incident in the early 1970s. At this time he had engaged in drug trafficking and got involved in an argument about a drug delivery that had not gone as planned. In the course of the argument, our patient suffered an abdominal gunshot wound with extensive injury. Unfortunately, there were no medical reports available from that time due to the patient very rarely visiting a doctor. However, the patient recalled that his spleen and diaphragm were severely damaged and needed surgical repair. Indeed, our CT scans showed that a splenectomy had been performed.

**Fig. 4:**
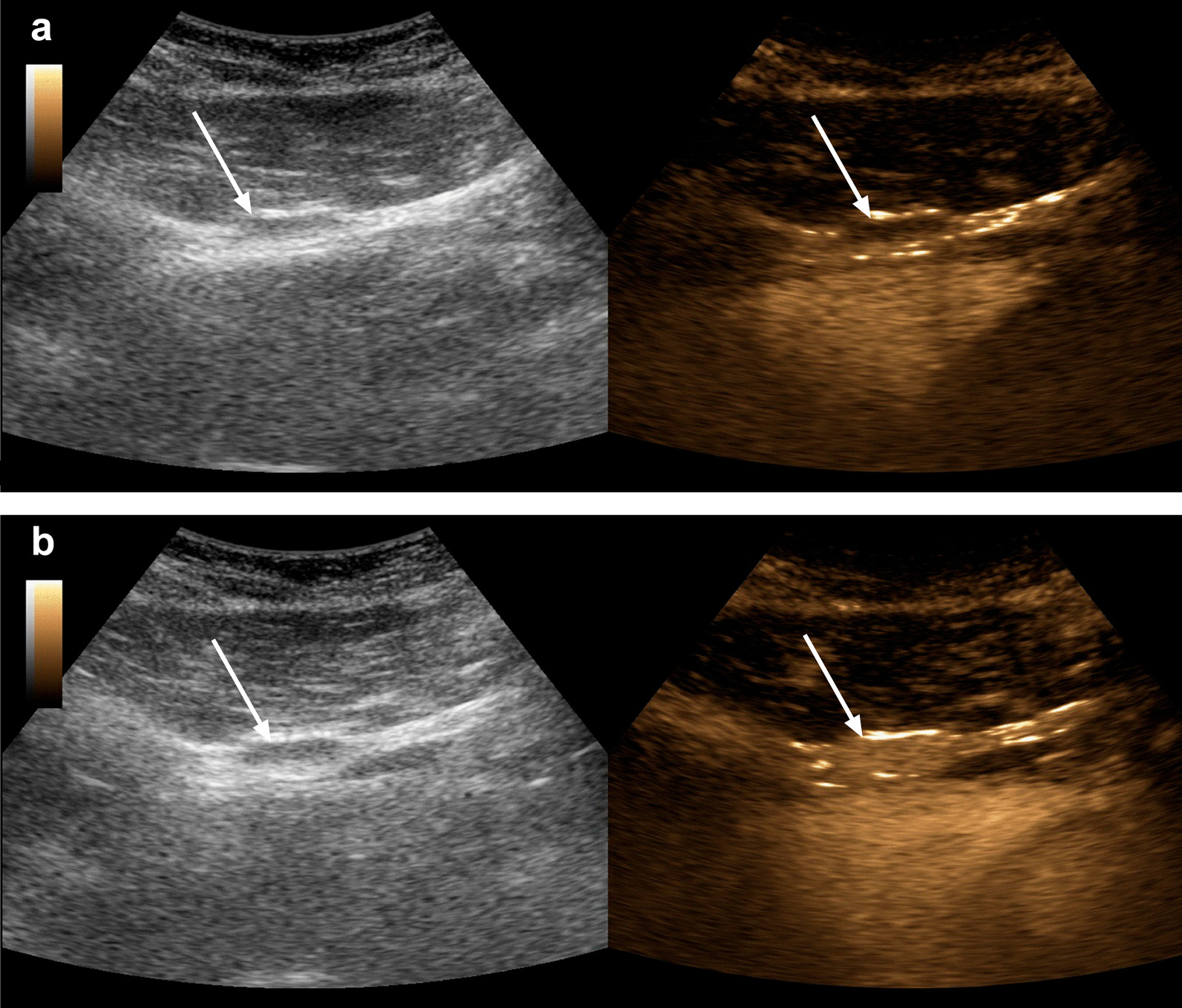
Contrast-enhanced ultrasound of a splenotic lesion between abdominal wall and liver. Images are taken before (**a**) and during persistent (> 4 minutes) late-phase contrast enhancement typical for splenic tissue (**b**)

Instead of biopsy, we then utilized contrast-enhanced ultrasound (CEUS) of the pleural and abdominal masses. On CEUS, a bolus of ultrasound contrast medium containing gas microbubbles is rapidly injected intravenously, followed by another bolus of saline solution for immediate dispersion via systemic circulation. When ultrasound waves are then directed at microbubbles flowing through the organ of interest, the compressible gas cores oscillate and show an increased echogenicity compared with surrounding tissue. Compared with other tissues, splenic tissue has the property to sequester microbubbles from circulation, causing an avid and persistent late-phase enhancement of up to 7 minutes after application, whereas malignant masses are characterized by early wash-out and late-phase hypoenhancement [7]. In our case, the application of sulfur hexafluoride microbubbles revealed circumscribed lesions that showed the characteristic pattern of splenic tissue, with persistent late-phase enhancement unlike malignant masses (Fig. 4a, b) [7]. Together with the very specific patient history and the medical imaging we performed, we were now convinced that we found the correct diagnosis and decided on a conservative approach without additional, invasive diagnostic measures. Follow-up examinations after 12 months showed no progress of the lesions, confirming the diagnosis of abdominal and thoracic splenosis based on detailed patient history and CEUS. Importantly, we were able to refrain from diagnostic biopsy, which otherwise might have led to serious complications considering the highly perfused splenotic tissue and the deranged coagulation due to hepatitis C-related liver dysfunction. Since splenosis is a benign condition, no specific treatment was necessary. Instead, treatment for hepatitis C and chronic obstructive pulmonary disease was initiated to improve his prognosis and the patient received oral pain medication, which resolved his pleurisy.

## Discussion

Due to its preferred occurrence in the abdominal cavity in up to 76% of severe splenic trauma requiring splenectomy [3], splenosis is most frequently confused with tumors of abdominal origin [4]. Rarely, and only after diaphragmatic damage, has thoracic splenosis been described [8, 9]. As shown here, splenosis may present as disseminated intraabdominal and intrathoracic lesions resembling mesothelioma, advanced malignant tumors, or possibly echinococcosis. Splenotic lesions are usually located in body cavities or on the surface of intraabdominal or intrathoracic structures. However, very few cases of intraparenchymal splenosis have been described, and are usually accompanied by preceding organ damage [4, 10, 11]. Common misdiagnoses of splenosis include intraabdominal lymphoma, hepatic, gastrointestinal or gynecological cancers, and endometriosis, and the consequences of misdiagnosis should not be underestimated.

The suspicion of a malignant tumor leads to stress for the patient and might cause a psychological burden, even when ultimately overturned. Moreover, diagnostic measures arising from the suspected diagnosis may lead to serious complications. The hemorrhagic potential of well-perfused and vulnerable splenotic masses is well documented by several case reports of spontaneous and posttraumatic bleeding in the peritoneal and thoracic cavities of patients with splenosis [12–15] implicating the necessity for careful evaluation of diagnostic biopsies to prevent complications. On the other hand, these considerations must never lead to delayed diagnosis of malignant tumors, since treatment at early stages leads to substantially improved outcomes compared with later stages. In our case, considering the deranged coagulation of our patient due to hepatitis C-related liver dysfunction, an attempted biopsy of one of the lesions might have led to severe bleeding. Additionally, nuclear imaging utilizing Tc-99m sulfur colloid or Tc-99m-tagged heat-damaged autologous erythrocytes are considered the gold standard to complete the diagnosis of splenosis, but have inherent risks due to radiation exposure. We here want to emphasize the importance of detailed patient history and CEUS as a noninvasive and affordable diagnostic technique that can be used to confirm the diagnosis when performed by an experienced examiner. However, biopsy should be considered when the patient history does not include injury to the spleen of any kind since this seems to be a prerequisite for most cases of splenosis [2, 3]. Moreover, if follow-up imaging reveals progressive lesions, histological examinations are necessary since malignant tumors need to be ruled out.

## Conclusion

We present a case of extensive intrathoracic and intraperitoneal splenosis mimicking malignant tumors in a patient that had suffered splenic trauma in the past. Suitable noninvasive diagnostic tools such as CT scans and CEUS performed by experienced examiners were used for diagnosis, and unnecessary invasive diagnostics were avoided. This case highlights that it is important to consider splenosis as a rare differential diagnosis for malignant tumors of the abdominal and thoracic cavities if there is a matching splenic trauma in the medical history. Thus, patients should be specifically asked for abdominal injuries and splenectomy in their medical history, even decades in the past. However, necessary diagnostics for malignant tumors must not be delayed, especially if there is no typical history of splenic trauma or the lesions are progressive. Follow-up controls and, if necessary, nuclear imaging can be useful to ultimately confirm the diagnosis.

## Data Availability

Not applicable.

## Abbreviations

CEUS: Contrast-enhanced ultrasound
CT: Computed tomography
